# Stem revision vs. internal fixation in Vancouver B2/B3 periprosthetic hip fractures: systematic review and metanalysis

**DOI:** 10.1007/s00402-024-05469-1

**Published:** 2024-08-06

**Authors:** Alberto Di Martino, Matteo Brunello, Eleonora Villari, Claudio D’Agostino, Monica Cosentino, Barbara Bordini, Fabrizio Rivera, Cesare Faldini

**Affiliations:** 1 I Orthopedic and Traumatology Department, IRCCS Istituto Ortopedico Rizzoli, Bologna, 40136 Italy; 2 Department of Biomedical and Neurimotor Science-DIBINEM, University of Bologna, Bologna, 40136 Italy; 3 Medical Technology Laboratory, IRCCS Istituto Ortopedico Rizzoli, Bologna, 40136 Italy; 4 Department of Orthopedic Trauma, SS Annunziata Hospital, Savigliano, 12038 Italy

**Keywords:** Periprosthetic fracture, Vancouver classification, B2, B3, THA revision, Fixation, ORIF

## Abstract

**Introduction:**

Vancouver B2 and B3 periprosthetic fractures (PFF) are associated with stem instability and often require a demanding stem implant revision (SR) or internal fixation (ORIF). This latter surgery is increasingly performed in the last few years instead of SR, but it is unclear which is the best treatment to manage PFF patients. The aim of this study is the compare the outcomes of B2/B3 PFF managed by either ORIF or SR, by performing a systematic review and meta-analysis of current literature.

**Materials and methods:**

Cochrane Database, PubMed, Google Scholar and MEDLINE were examined to find out relevant publications dealing with the different outcomes of SR vs. ORIF in B2/B3 PFF of the hip. The effect model (EM) was calculated using Cohen´s d index.

**Results:**

Fifteen studies were included, reporting on a total of 1629 patients (564 ORIF and 1065 SR). The pooled random EM estimates for reoperation was 0.87 (95% CI, 0.39–1.96; I2 = 78%) in favor of ORIF surgery; EM for complications was 1.01 (95% CI, 0.45–2.27; I2 = 85%) without difference among procedures. The EM for transfusion was 0.72 (95% CI, 0.46–1.12; I2 = 62%) in favor of fixation.

**Conclusion:**

ORIF and SR were both suitable and effective options in PFF patients, being associated to similar complications rates. Our results show that ORIF performance in PFF patients is associated to significantly less in blood loss, surgical time and in-hospital stay. These advantages are particularly appealing in patients with multiple comorbidities.

## Introduction

**Fig. 1:**
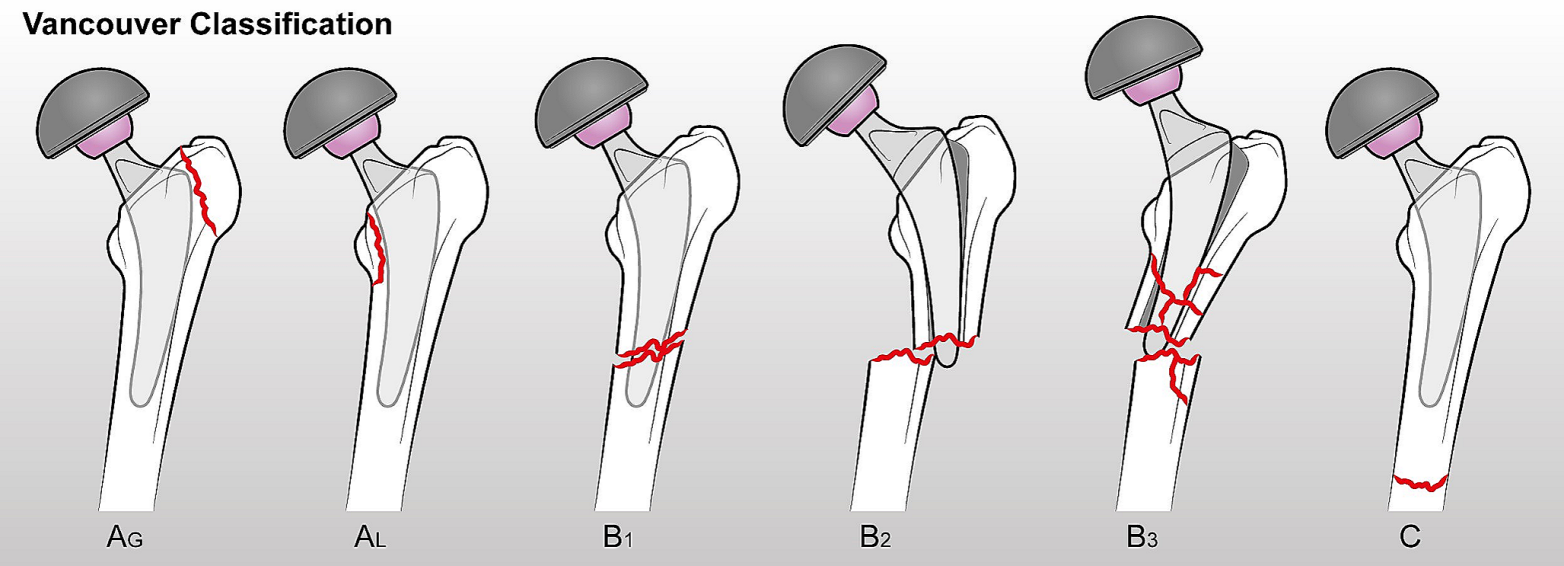
Vancouver Classification of periprosthetic implant fractures at the hip. Type **A**: Fracture in the trochanteric region, involving Lesser (AL) or Greater (AG) trochanter. Type **B**: B1 Fracture around stem or just below, with well-fixed stem, B2 Fracture around stem or just below, with loose stem but good proximal bone, B3 Fracture around stem or just below, with loose stem and poor quality or severely comminuted proximal bone. Type **C**: Fracture below the stem of the implant, with stability of the implant

Periprosthetic fracture of the femur (PFF) is one of the major complications occurring during or after total hip arthroplasty (THA). When these occur in the post-operative period, PFF is the third principal cause of THA failure requiring surgical revision, after aseptic loosening and recurrent dislocation [1–4], ranging from 1 to 11% in primary implants [5]. The Vancouver classification is the most widely used to guide fracture diagnosis and management, dividing PFF into two main categories: intraoperative and postoperative. These categories are further classified in A, B or C according to fracture location (Fig. 1): A types are located at the proximal metaphysis without extending into the diaphysis, and these are further subdivided into those involving the greater (AG) and lesser (AL) trochanter; B types affect the femoral diaphysis around the stem, and quality and stability of stem implant and residual proximal femoral bone stock are used to divide B group into three subcategories: in type B1 fractures the stem is well fixed, in type B2 there is a loose stem with good bone stock and in type B3 the stem is loose and the surrounding bone stock is poor. C types are located below the tip of the stem of the implant [6].

Vancouver B2 and B3 types are the most commonly encountered in clinical practice, being reported in 75–85% of all PFF [7]. These fractures are considered “unstable” subtypes because the fracture develops around the stem itself, making the treatment particularly challenging. Current recommendations for surgical treatment of Vancouver subtypes B2 and B3 is revision with a long femoral stem to bypass the fracture site, with or without associated internal fixation [8]; this technique is associated to a lower risk of failure [9]; however, some Authors reported poor results and a higher perioperative mortality rate when stem revision procedures are performed in this patients population, suggesting that a less invasive reduction and internal fixation, when possible, might be a suitable option. At present, internal fixation is reserved for more fragile patients [10, 11] because of the higher risk of fracture non-union in the presence of a loose implant, and for the requirement of a longer rehabilitation [12]; however, when the fracture heals properly, there is no requirement for later implant revision and the patients may benefit from a surgical procedure of reduced complexity, offering similar outcomes compared to revision arthroplasty [13]. This technique might result in several benefits including shorter surgical time and reduced blood loss, and it could be particularly helpful in elderly and fragile patients in which a more demanding revision arthroplasty surgery may adversely affect the overall prognosis and patients’ survival [14].

The aim of this study is therefore to compare in a systematic literature review and meta-analysis the outcomes of stem revision (SR) procedure vs. open reduction and internal fixation (ORIF) in patients affected by Vancouver B2 or B3 periprosthetic fractures.

## Materials and methods

### Literature search

**Fig. 2:**
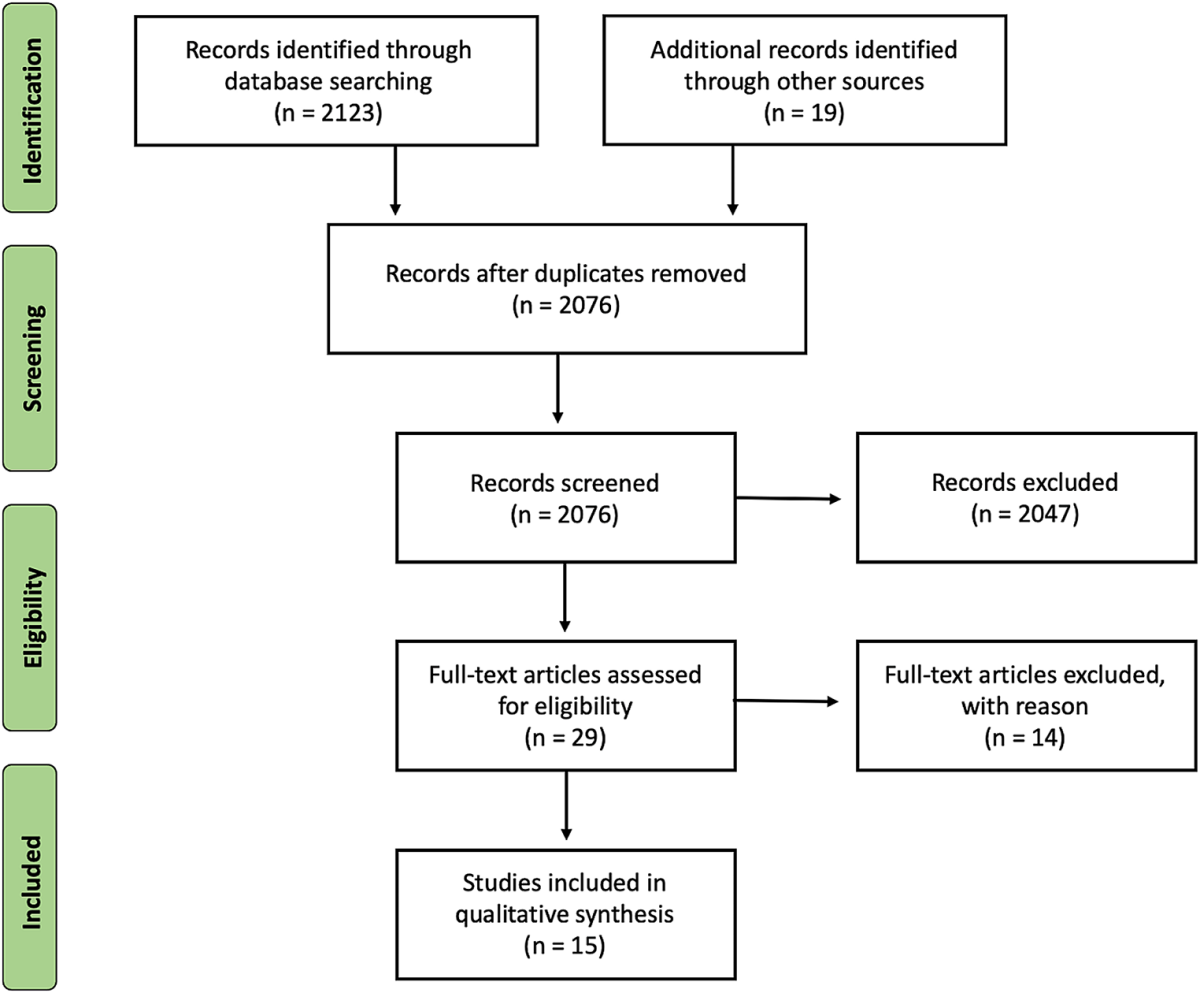
Prisma flowchart

On April 15th 2023, the Cochrane Database of Systematic Reviews and the medical databases PubMed, Google Scholar and MEDLINE were examined to find relevant publications on the different outcomes using stem revision versus fixation in patients with periprosthetic Vancouver B2/B3 hip fractures. A patient intervention compared outcome of interest (PICO) model was used. Studies published in English between June 1984 and April 2022 were included from the databases. The search was performed using the terms “femoral”, “fracture”, “hip” and “periprosthetic”. Then, to identify other works relevant to the subject, references of pertinent publications were examined. Articles describing the different outcomes using stem revision versus osteosynthesis fixation in periprosthetic hip fracture Vancouver B2/B3 were included. However, articles were excluded if published in a language other than English, or if describing outcomes of cohorts of less than fifty patients. Two authors (MB, EV) independently evaluated and screened the titles and abstracts of the identified papers according to our inclusion and exclusion criteria, the articles deemed to be of interest were then chosen for a full text analysis. In accordance with the Preferred Reporting Item for Systematic Reviews and Meta-Analyses (PRISMA) criteria, a flowchart was made to illustrate the steps involved in include the reviewed works (Fig. 2).

### Qualitative assessment and risk of bias

Two authors (BB, LT) independently evaluated the strength of the evidence and the caliber of each study using the methodological index for non-randomized studies (MINORS). It evaluates 12 criteria, the first 8 of which were expressly created to assess the caliber of research in non-randomized studies. These include a clear statement of the study purpose, the inclusion of consecutive patients, prospective data collection, and the appropriateness of the endpoint to the goal of the study, which should be unbiased. Moreover, the study follow-up should be explicitly reported and being appropriate for the goal of the study, with a loss of less than 5% of patients at follow-up. The material and methods section of the study should also include a prospective computation of the study size. Each component receives a score between 0 and 2: 0 for non-reporting, 1 for poor reporting, and 2 for satisfactory reporting. For non-comparative studies, a figure of 16 is the best available score. To assess the risk of bias within the included studies, their methodological quality was assessed by using the Grading of Recommendations, Assessment, Development, and Evaluations (GRADE) cohort checklist for assessing the quality of non-randomized studies.

### Data extraction and report

The selected studies were analyzed, and the information of interest was extracted onto a database created using Microsoft Excel for Microsoft Mac (Microsoft Corporation, Redmond, Washington, USA). The following data were recorded, if available: number and age of patients, type of fracture (B2/B3), BMI, time from THA to fracture, clinical outcomes by hip functional scores, surgical time, surgical approach, type of fixation including the use of bone cement, reoperation rate, postoperative complications, blood loss, average hospital stay and length of follow-up. The extracted data were reported by descriptive statistics. Continuous variables were reported as average and range (minimum–maximum). Categorical variables were reported as frequencies and percentages.

### Outcome measures

Measured outcomes included the type of surgical procedure used in ORIF and SR. Data on surgical time, in hospital stay and perioperative blood loss were retrieved from selected manuscripts. The complication rate related to each surgical procedure was indagated, including surgical and not surgical complications, aseptic and septic loosening, intraoperative fractures, wound dehiscence, dislocation, hematoma, etc. Revision rate included all reasons that led to failure and required surgical stem implant revision, including aseptic loosening, dislocation, isolated fracture or periprosthetic fracture. Moreover, functional scores pre- and post-operatively were recorded and compared, analyzing the Parker Mobility Score (PMS) and the Harris Hip Score (HHS).

### Statistical analysis

Primary grouping for analysis was based on the type of surgical indication in periprosthetic Vancouver B2/B3 hip fractures. The endpoints that were evaluated included reoperation, complication, transfusion, surgical time and hospital stay. To assess the effect of stem revision vs. internal fixation on dichotomous variables, weighted risk ratios (RR) were calculated to pool study and control groups in each publication for analysis. Random effects models were used regardless of the heterogeneity assessments. Forest plots of the SMD or RR were generated with all studies. The I2 statistic was used to help assess heterogeneity. Fixed effects models were used for sensitivity analyses. R Core Team (2023). R: A Language and Environment for Statistical Computing. R Foundation for Statistical Computing, Vienna, Austria. <https://www.R-project.org/>. software was used to perform the analyses [15, 16].

## Results

### Search results

As shown on the PRISMA FLOWCHART (Fig. 2) 2076 manuscripts were found at the initial search. 2047 studies were eliminated from consideration based on their title and abstract, because these were deemed unrelated to the current investigation. To ensure that the remaining 29 manuscripts met the inclusion criteria, a thorough evaluation was conducted: 15 studies satisfied the inclusion criteria after full text screening, with a total of 1629 recruited patients, 564 managed by ORIF and 1065 by SR. 

### Study quality assessment

All the selected studies were retrospective case series of medium to large size (N range 51–278 patients) reporting the outcomes of SR vs. ORIF in PFF classified as Vancouver B2 and B3. All retrieved manuscripts were published between 2006 and 2023. According to the MINORS evaluating score system, on a maximum of 16 points, 1 study achieved 8 points [17], 1 study achieved 10 points [18], 2 studies achieved 12 points [19, 20], 5 studies achieved 13 points [21–25], 4 studies achieved 14 points [26–29], 1 study achieved 15 points [30] and 1 study reached 16 points [31].

### Cohort characteristics

The studies included 1629 patients with a mean age of 77 years (range 66.8–87); average follow-up was 44.4 months (range 6–78 months). The median BMI was 25.4. Considering the study population according to Vancouver B2 or B3 type, our work featured 1128 B2 and 333 B3 fractures.

#### Surgical technique

Revision surgery consisted of stem revision by longer stem implant with or without the use of plate and/or cable wires, regardless of the use of bone cement; internal fixation included the use of locking compression plate (LCP), in some cases combined with Locking Attachment Plate (LAP), and limited contact dynamic compression plate (LC-DCP), with or without the use of cable wires. Surgery was performed by direct lateral approach in all the studies that reported surgical approach [21, 22, 25, 26, 28].

#### Complications and mortality

10 of the 15 studies reported complications, distinguishing among ORIF and SR [17, 20, 21, 25, 26, 28–32], reporting data on 979 patients (437 ORIF and 542 SR). More specifically, 7 of the 15 studies [17, 20, 21, 26, 28, 29, 32] reported subgroups of complication in a total of 629 patients, 288 treated with ORIF and 341 treated with SR. 23 out of 288 (7.9%) ORIF patients developed medical complications and 30/288 (10.4%) developed implant-related complications. 32 out of 341 (9.3%) SR patients developed medical complications and 42/341 (12.3%) developed implant-related complications. The most frequently reported postoperative complication was infection (14/288, 4.8% in ORIF group vs. 18/341, 5.2% in SR group), followed by aseptic loosening (13/288, 4.5% in ORIF vs. 6/341, 1.7% in SR), dislocation (4/288, 1.4% in ORIF vs. 14/341, 4.1% in SR), malunion (7/288, 2.43% in ORIF vs. 3/341, 0.9% in SR), refracture (3/288, 0.9% in ORIF vs. 6/341, 1.7% in SR) and implant rupture (1/288, 0.3% in ORIF vs. 1/341, 0.3% in SR). As regards mortality in the first postoperative year, 17/288 (5.9%) cases were reported in ORIF group and 14/341 (4.1%) in the SR group.

#### Hip function scores

10 of the 15 studies reported function scores [17, 18, 20–22, 25, 26, 28–30], describing a general improvement from preoperative to postoperative condition. The Parker Mobility Score (PMS) [33] was the most commonly utilized [18, 21, 25, 28, 29], followed by the Harris Hip Score (HHS) [34]. In the studies where PMS was used, patients treated with ORIF had an average of 4.2 PMS score while SR group had a 6.3 PMS score (*p* = 0.022). The evaluation with the post-operative HHS showed better results of SR compared to ORIF (HHS 67.5 for O vs. 74.8 for R), although not statistically significant (*P* = 0.148).

### Meta-analysis and risk of bias

**Fig. 3:**
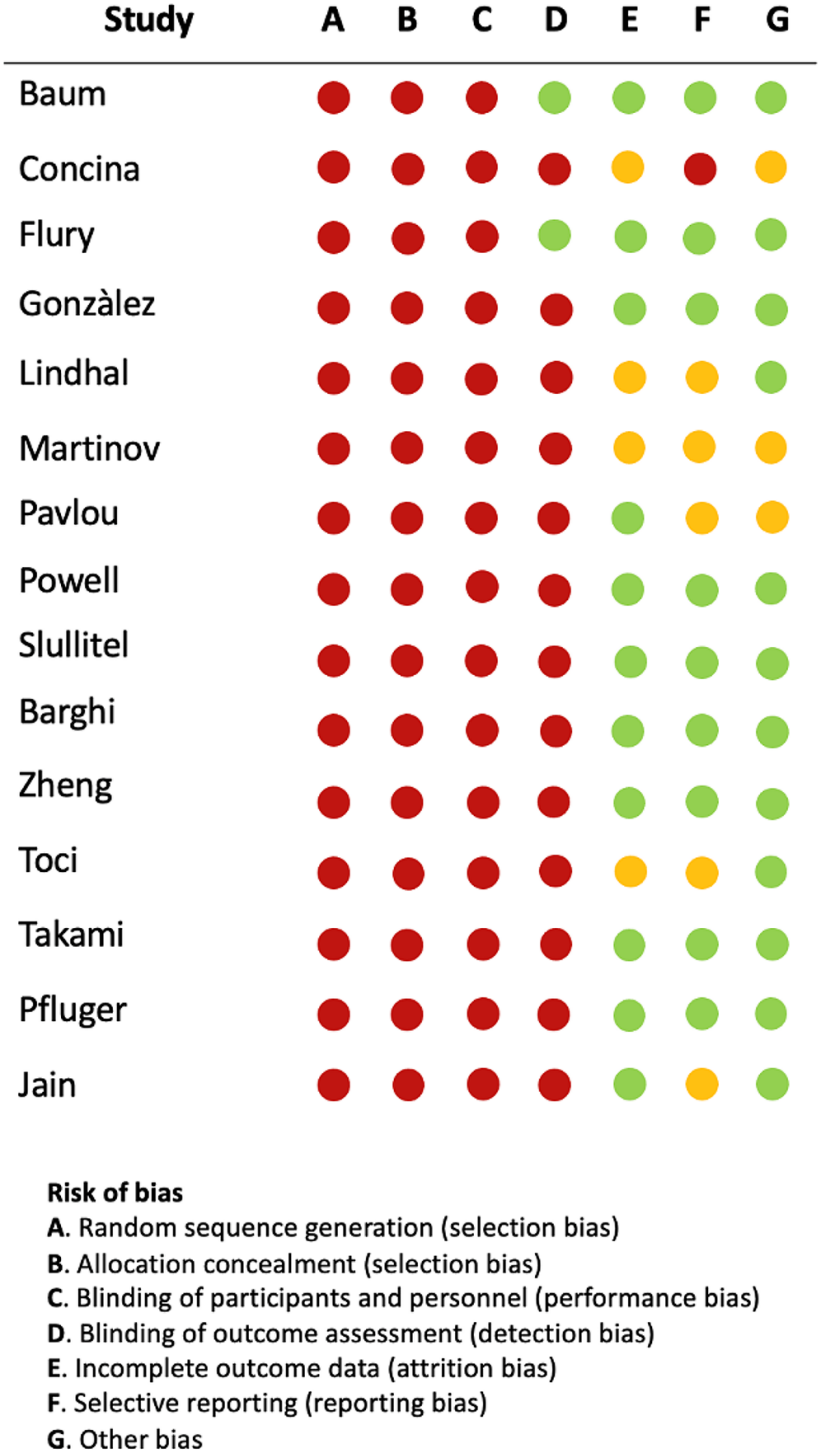
Risk of bias assessment for included studies (GRADE)

Risk of bias was assessed for each individual study (Fig. 3). Fifteen studies were amenable for being included in the meta-analysis.

**Fig. 4:**
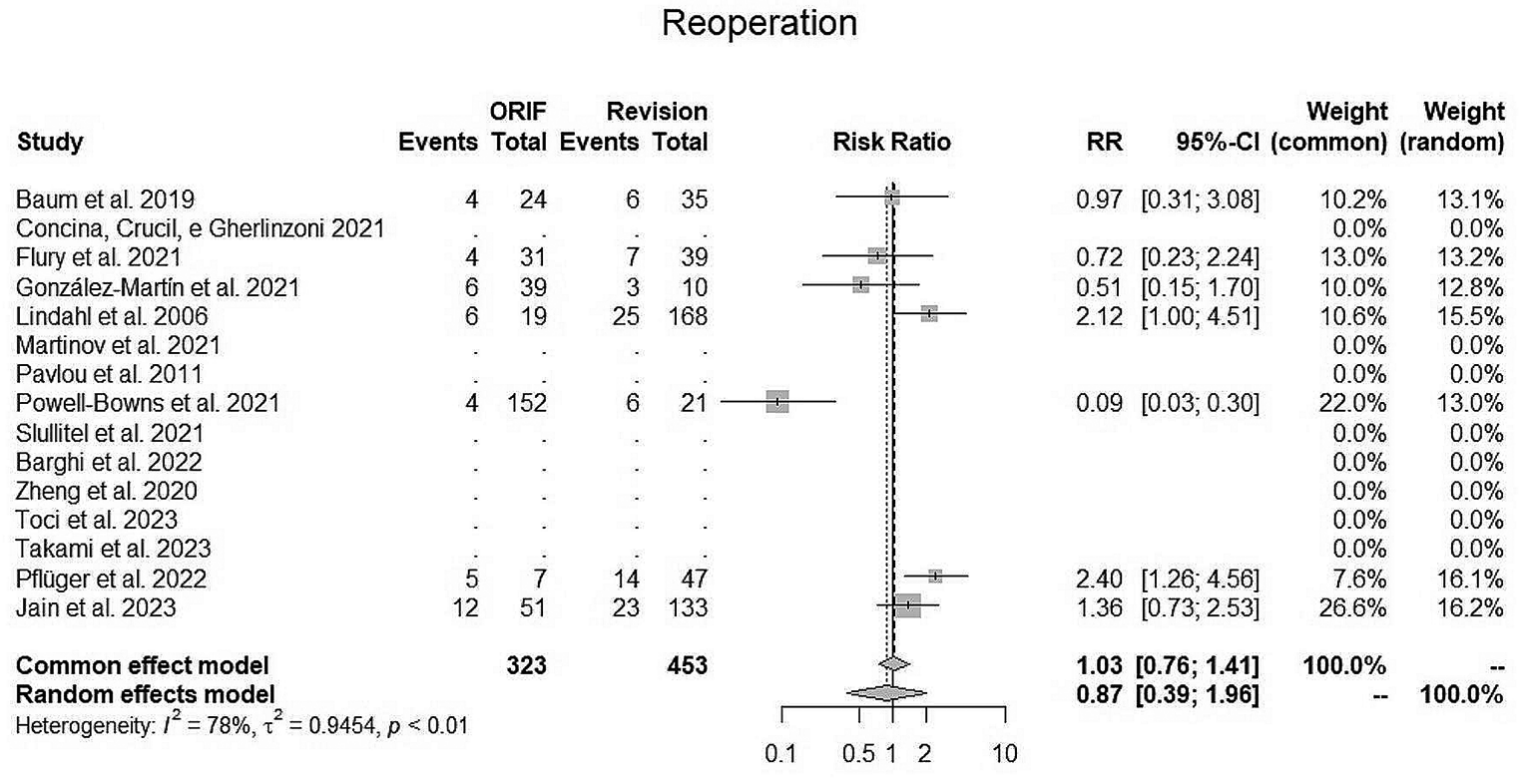
Reoperation

The pooled random EM estimated for the **reoperation** (Fig. 4) was 0.87 (95% CI, 0.39–1.96; I2 = 78%) for fixation surgery, showing a lower reoperation rate in favour of ORIF procedure. In fact, out of 323 patients treated with fixation only 41 needed reoperation (12.7%), while in the SR group 84 out of 453 patients (18.5%) required re-operation.

**Fig. 5:**
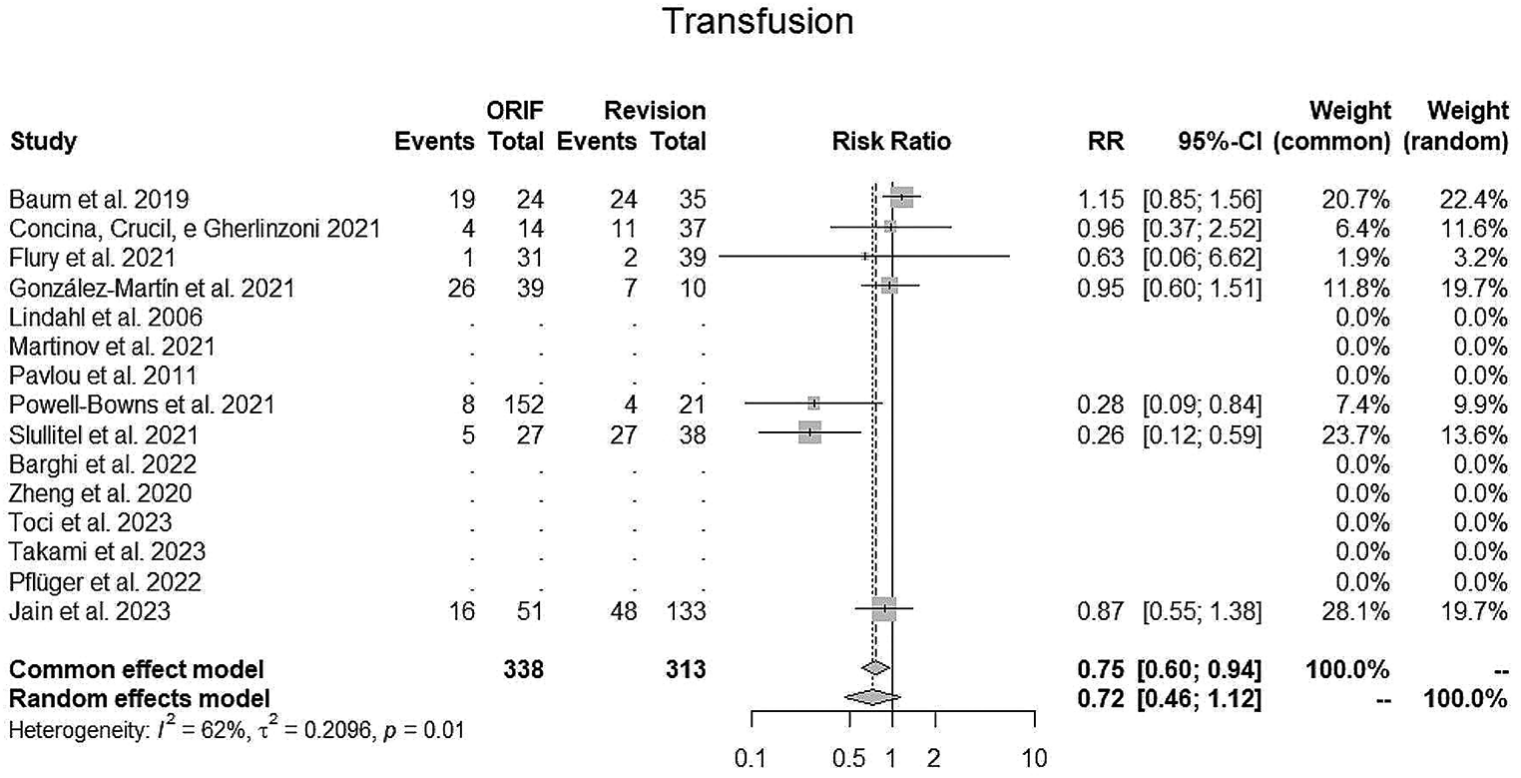
Complications

As regards the overall **complications** (Fig. 5) the random EM was 1.01 (95% CI, 0.45–2.27; I2 = 85%) for revision surgery; however, EM value in this case suggests similar outcomes in both groups. In the 10 studies reporting data from complications, 6 showed lower rates for ORIF [17, 22–24, 26, 27], and 4 had a lower rate of complications when SR was performed. 78/437 (17.8%) ORIF patients developed complications compared to 117/542 (21.5%) patients in SR group.

**Fig. 6:**
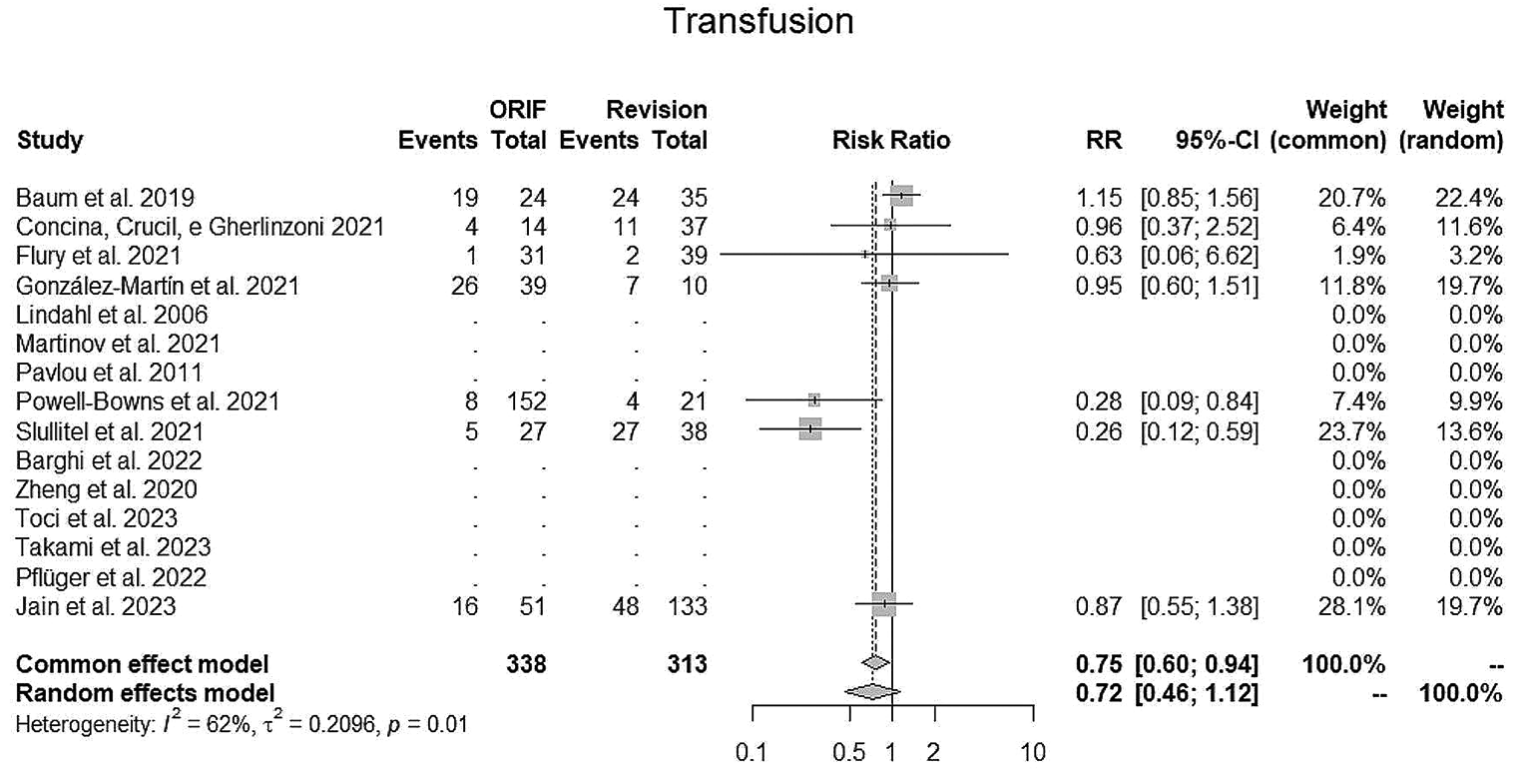
Trasfusions’ rate

The random EM for **transfusion** (Fig. 6) was 0.72 (95% CI, 0.46–1.12; I2 = 62%) in favour for fixation surgery.

**Fig. 7:**
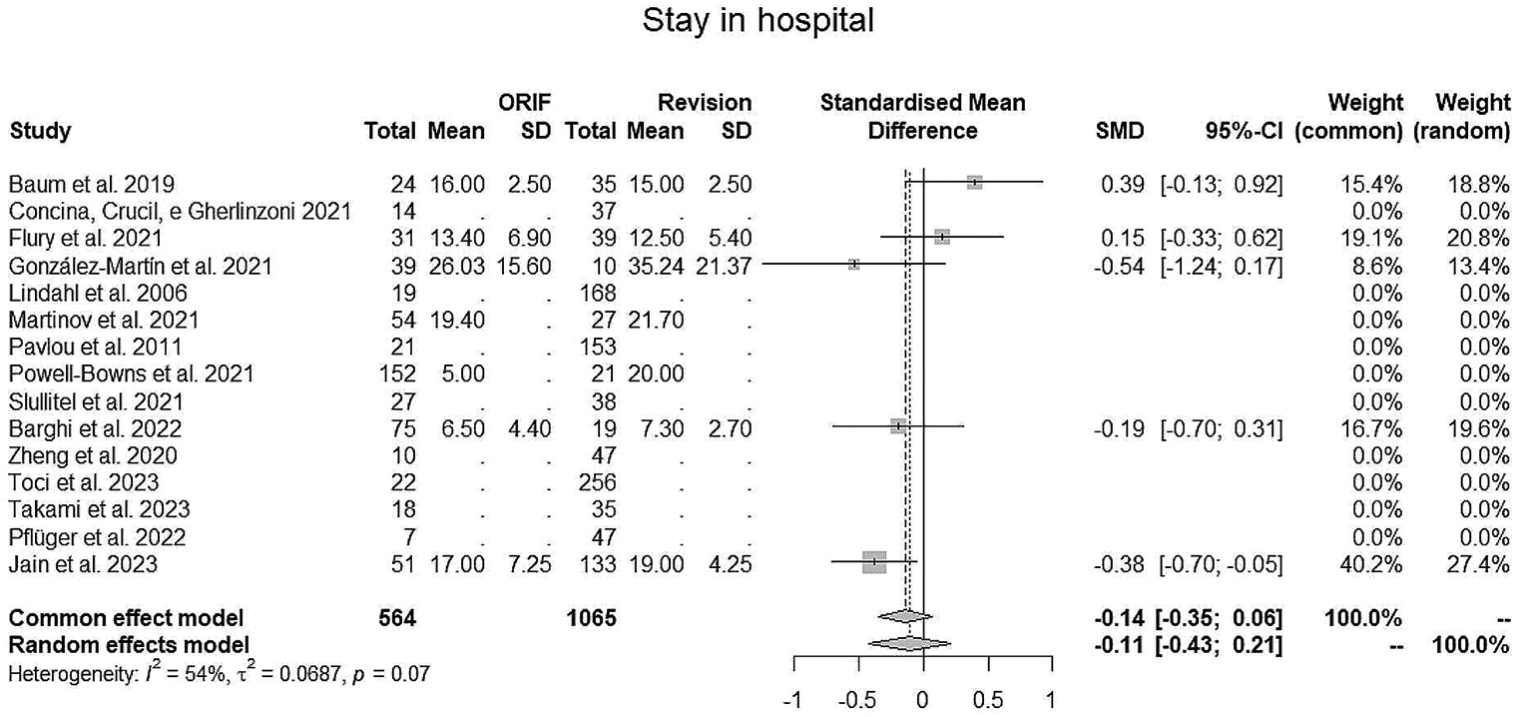
Stay in hospital

**Fig. 8:**
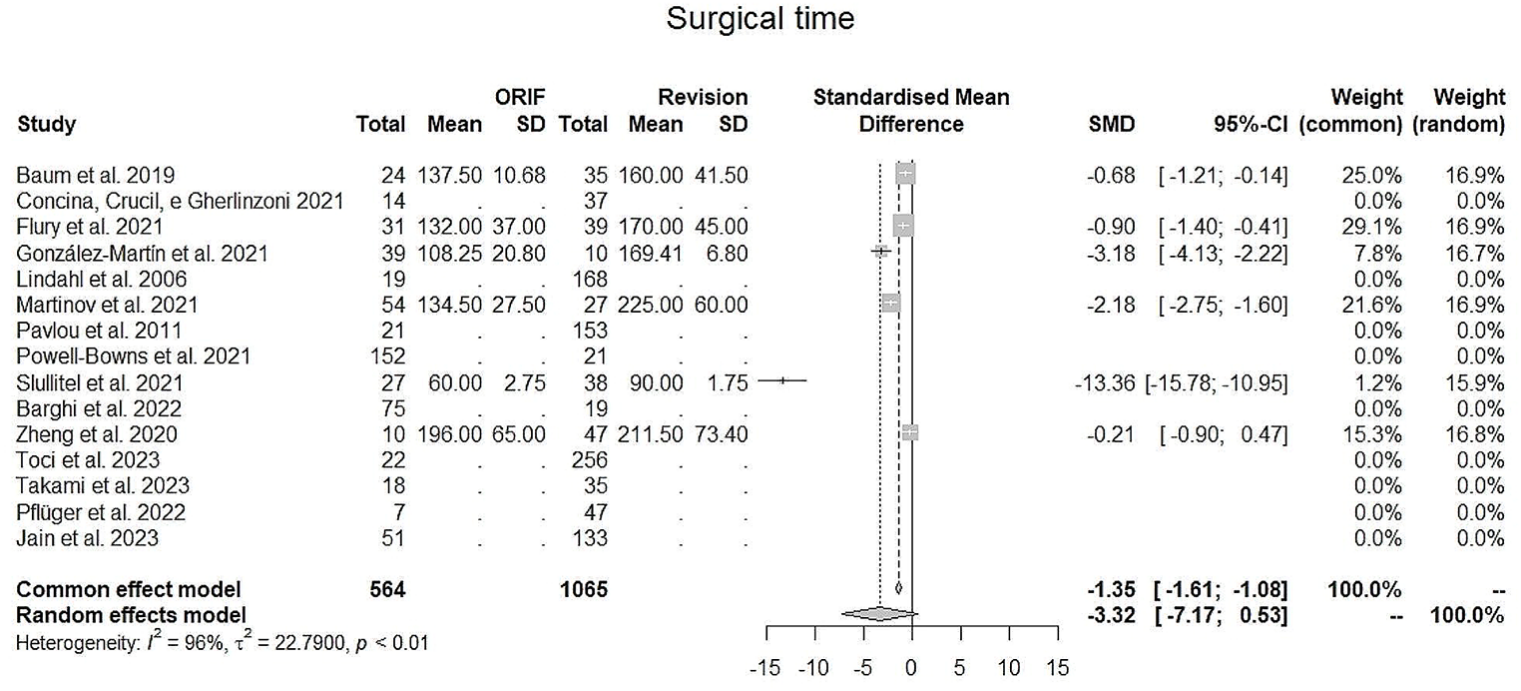
Surgical time

The evaluation for **in hospital stay** (in days) (Fig. 7) and **surgical time** (in minutes) (Fig. 8) showed better outcomes for ORIF, with a reduced time for both parameters. The ORIF group registered an average hospital stay of 12.6 days, versus 18 of the stem revision group. Regarding surgical time, ORIF required an average 129.3 min, while SR required mean 161.2 min. It was not possible to perform an analysis on cemented vs. uncemented and collar use due to lack of clarity and data within the papers.

## Discussion

The most important finding of the current study is that in Vancouver B2/B3 periprosthetic fractures of the hip, ORIF surgery obtained better results compared to SR in several evaluated outcomes, showing a lower re-operation rate, reduced surgical time and in hospital stay, with a lower requirement for blood transfusion. Overall complications showed similar results in both procedures. In PFF patients, SR procedures showed superior outcomes in terms of fracture union rates and long-term functional results.

Analyzing recent literature, only three meta-analysis are available on the current topic; two were released in 2021 and 2022 [35, 36], and these showed that PPF management by SR was associated to a reduced risk in reoperation and revision rate. In the meta-analysis by González‑Martín [37], unclear results about the superiority of ORIF treatment over SR were reported, and the Authors supported the use of ORIF in selected patients with low functional request and multiple comorbidities.

PFF patients are typically associated with poor functional outcomes; therefore, to better understand of the clinical conditions, several measures are used to evaluate overall function before and after treatments: these scores include HHS, PMS, OHS, PROMIS and VAS pain assessment, and these provide standardized ways to assess pain levels, functional ability, and overall patient satisfaction [38, 39]. In the studies in which PMS was evaluated [18, 21, 25, 28, 29], there were better postoperatively functional outcomes for the patients treated by SR compared to ORIF; similar findings were obtained observing the post-operative HHS in the two groups [17, 20, 22, 29], Moreover, the authors reported similar outcomes also for pain, stating that SR patients could better manage postoperative pain [18, 20, 29, 30]. Most probably, these results can be attributed to the a priori patient selection in retrospective the cohorts; in fact, ORIF was reserved for patients with comorbidities and compromised general health status [18, 25], while SR was performed in younger and more healthy patients.

Overall revision rate was higher in SR group. A total of 7 manuscripts included this parameter, three showing better reoperation rate with SR [20, 23, 31] and 4 supporting ORIF [21, 26–28]. Powell et al. [27] et al. in his study showed a very low rate (4/152) of reoperation in ORIF patients. Conversely, Lindhal et al. [23] in a cohort of 153 SR and 21 ORIF, showed better outcomes in the revision group. These contrasting results that favour SR or ORIF are most probably related to the dedicated expertise of the institution where the surgery was performed, whether it was a trauma or an arthroplasty facility.

In the current metanalysis, the overall complication rate was similar between the two groups of treatments. In literature complications are reported after surgery for PFF in up to 48% of patients [40]. The risk of complications in PPF of the hip is a significant concern; SR and ORIF showed similar complication rates, confirming data from literature [17, 18, 29]. Therefore, the choice of treatment most probably requires balance of the patient’s overall health status, and the available expertise at the treating centre [8].

Patients affected by hip PPF, are typically affected by several comorbidities [41] and show poor bone quality and low body muscle mass [42]. Seen the fragility of PPF patients, the estimated surgical time and blood loss are of critical importance to perform a safe surgery minimizing intra and post-operative risks [18]. The analysis of the included studies suggests that ORIF for PPF may require less transfusions compared to SR. Several studies reported lower transfusion rates in ORIF patients [21, 22, 24–28, 30, 31]. Common sense suggests that the finding is due to the less invasive nature of ORIF procedures, which generally involve smaller surgical exposures and limited bone and soft tissue dissection. The preservation of the native femoral stem in osteosynthesis may also contribute to the reduced blood loss as compared to the more extensive exposure required in SR surgery [20]. Furthermore, ORIF surgery is typically associated to a reduced surgical time [21, 25, 26, 28] compared to SR. Martinov et al. [24] in a study of 90 patients treated by either procedures found that surgical time in SR patients almost doubles ORIF (134 vs. 225 min).

The limited blood loss and shorter surgical time of PPF patients managed by ORIF are factors determining the reduced in-hospital stay for patients with PPF managed by osteosynthesis compared to implant revision [27, 29, 31]. Zheng et al. [29] confirmed this finding; in their study, they also investigated the time in Intensive Care Unit for ORIF and SR procedures, and found an average permanence of 0.2 days in PPF patients operated by osteosynthesis compared to an average 0.7 days for SR.

It is important, when discussing about periprosthetic fractures, to understand whether the implant is cemented or not, and whether modular collars are used in the implants. These factors can influence the survival of the implants.

In fact both present advantages, cemented stems provide stability through controlled subsidence within a cement mantle, which stabilizes upon cement polymerization. Some author recommend ORIF alone for cemented stems with periprosthetic fractures if the cement–bone interface remains intact and only the cement-stem interface is disrupted by the fracture [13]. Managing fractures around cemented implants with ORIF is often more technically challenging because both the bone-cement and cement-stem interfaces contribute to implant stability. Additionally, patients with cemented implants frequently have poor bone quality and may ultimately require revision arthroplasty if ORIF alone cannot restore stem stability after a periprosthetic fracture, further complicating the case [30].

Uncemented implants are easier to revise and do not carry a risk of cement extrusion into the fracture site or interference on fracture healing, leading to non-union. Uncemented, extensive coated prostheses have been shown to perform better than cemented stems for revision in type B (B1-B3) fractures. Disadvantages include limited weight-bearing in the immediate post-operative period, stress shielding and stem subsidence [43].

Currently, there are few studies in the literature on this topic, and their results are sparse and limited, but the data present reported that there was no significant difference in the reoperation rate and survival following use of the cemented stems and use of the uncemented stems [7, 44].

Our study, like many systematic reviews and meta-analyses, has several limitations. These include the presence of selection bias, as well as the restricted availability of complete information from the included reports. Additionally, there is a possibility of language selection bias since only studies published in English were included, potentially excluding relevant studies published in other languages. It is important to acknowledge these limitations because these may impact the chance to generalize and extend our findings to the general population. However, the decision to include only studies reporting at least 50 patients gives strength to our findings because only outcomes from high volume surgical centres were included. Finally, greater surgical expertise towards revision or ORIF is a factor that can certainly influence the results. This factor is not identifiable and is not described in the papers that can be selected in the literature.

In conclusion, ORIF is a reliable alternative to SR to manage instable periprosthetic fractures around hip stems; it is associated to a reduced rate of reoperations, transfusion, surgical time and in-hospital stay, making it more suitable for patients with reduced functional requests and worse general health status. SR is associated to better functional outcomes, and it should be mainly considered in PFF patients with less comorbidities.

## Data Availability

All the data are present in the manuscript.

## References

[1] Sidler-Maier CC, Waddell JP (2015) Incidence and predisposing factors of periprosthetic proximal femoral fractures: a literature review. Int Orthop (SICOT) 39:1673–1682. 10.1007/s00264-015-2721-y 10.1007/s00264-015-2721-y 25813458

[2] Di Martino A, Pederiva D, Bordini B et al (2022) Proximal femoral replacement for non-neoplastic conditions: a systematic review on current outcomes. J Orthop Traumatol 23:18. 10.1186/s10195-022-00632-z 35348913 10.1186/s10195-022-00632-z PMC8964877

[3] Zhang Y, Gao Z, Zhang B et al (2022) The application of custom-made 3D-printed titanium augments designed through surgical simulation for severe bone defects in complex revision total hip arthroplasty. J Orthop Traumatol 23:37. 10.1186/s10195-022-00656-5 35932367 10.1186/s10195-022-00656-5 PMC9357241

[4] Di Martino A, Bordini B, Geraci G et al (2023) Impact of previous lumbar spine surgery on total hip arthroplasty and vice versa: how long should we be concerned about mechanical failure? Eur Spine J. 10.1007/s00586-023-07866-3 37498345 10.1007/s00586-023-07866-3

[5] Capone A, Congia S, Civinini R, Marongiu G (2017) Periprosthetic fractures: epidemiology and current treatment. Clin Cases Min Bone Metab 14:189–196. 10.11138/ccmbm/2017.14.1.189 10.11138/ccmbm/2017.14.1.189 PMC5726208 29263732

[6] Rayan F, Dodd M, Haddad FS (2008) European validation of the Vancouver classification of periprosthetic proximal femoral fractures. J Bone Joint Surg Br 90:1576–1579. 10.1302/0301-620X.90B12.20681 19043127 10.1302/0301-620X.90B12.20681

[7] Lindahl H, Malchau H, Herberts P, Garellick G (2005) Periprosthetic femoral fractures classification and demographics of 1049 periprosthetic femoral fractures from the Swedish National Hip Arthroplasty Register. J Arthroplasty 20:857–865. 10.1016/j.arth.2005.02.001 16230235 10.1016/j.arth.2005.02.001

[8] Mondanelli N, Troiano E, Facchini A et al (2022) Treatment algorithm of Periprosthetic Femoral Fracturens. Geriatr Orthop Surg Rehabil 13:21514593221097608. 10.1177/21514593221097608 35573905 10.1177/21514593221097608 PMC9096211

[9] Khan T, Grindlay D, Ollivere BJ et al (2017) A systematic review of Vancouver B2 and B3 periprosthetic femoral fractures. Bone Joint J 99–B:17–25. 10.1302/0301-620X.99B4.BJJ-2016-1311.R1 28363890 10.1302/0301-620X.99B4.BJJ-2016-1311.R1

[10] Stoffel K, Blauth M, Joeris A et al (2020) Fracture fixation versus revision arthroplasty in Vancouver type B2 and B3 periprosthetic femoral fractures: a systematic review. Arch Orthop Trauma Surg 140:1381–1394. 10.1007/s00402-020-03332-7 32086558 10.1007/s00402-020-03332-7 PMC7505881

[11] Brunello M, Di Martino A, Ruta F et al (2023) Which patient benefit most from minimally invasive direct anterior approach total hip arthroplasty in terms of perioperative blood loss? A retrospective comparative study from a cohort of patients with primary degenerative hips. Musculoskelet Surg. 10.1007/s12306-023-00792-z 37314642 10.1007/s12306-023-00792-z PMC10709233

[12] Di Martino A, Brunello M, Pederiva D et al (2023) Fast track protocols and early Rehabilitation after surgery in total hip arthroplasty: a narrative review. Clin Pract 13:569–582. 10.3390/clinpract13030052 37218803 10.3390/clinpract13030052 PMC10204442

[13] Solomon LB, Hussenbocus SM, Carbone TA et al (2015) Is internal fixation alone advantageous in selected B2 periprosthetic fractures? ANZ J Surg 85:169–173. 10.1111/ans.12884 25308044 10.1111/ans.12884

[14] Canton G, Rasio N, kristan A et al (2022) Should age be a factor in treatment choice of periprosthetic Vancouver B2-B3 proximal femur fractures? A retrospective analysis of mortality and functional outcomes in elderly patients. Acta Biomed Atenei Parmensis 92:e2021581. 10.23750/abm.v92iS3.12581 10.23750/abm.v92iS3.12581 PMC9437666 35604253

[15] Viechtbauer W (2010) Conducting Meta-analyses in R with the metafor Package. J Stat Softw 36:1–48. 10.18637/jss.v036.i03

[16] Balduzzi S, Rücker G, Schwarzer G (2019) How to perform a meta-analysis with R: a practical tutorial. BMJ Ment Health 22:153–160. 10.1136/ebmental-2019-300117 10.1136/ebmental-2019-300117 PMC10231495 31563865

[17] Pavlou G, Panteliadis P, Macdonald D et al (2011) A review of 202 periprosthetic fractures - stem revision and allograft improves outcome for type B fractures. HIP Int 21:021–029. 10.5301/hip.2011.6301 10.5301/hip.2011.6301 21298624

[18] Takami H, Takegami Y, Tokutake K et al (2023) Mortality and clinical outcomes of Vancouver type B periprosthetic femoral fractures: a multicentre retrospective study. Bone Jt Open 4:38–46. 10.1302/2633-1462.41.BJO-2022-0145.R1 36647618 10.1302/2633-1462.41.BJO-2022-0145.R1 PMC9887342

[19] Toci GR, Stambough JB, Martin JR et al (2023) Effect of fracture type, treatment and surgeon training on reoperation after Vancouver B Periprosthetic Femur fractures. J Arthroplasty S0883540323002553. 10.1016/j.arth.2023.03.024 10.1016/j.arth.2023.03.024 36933681

[20] Pflüger P, Bolierakis E, Wurm M et al (2022) Revision rate is higher in patients with periprosthetic femur fractures following revision arthroplasty in comparison with ORIF following our algorithm: a two-center 1 analysis of 129 patients. Eur J Trauma Emerg Surg 48:1913–1918. 10.1007/s00068-021-01832-8 34767064 10.1007/s00068-021-01832-8 PMC9192397

[21] Baum C, Leimbacher M, Kriechling P et al (2019) Treatment of Periprosthetic femoral fractures Vancouver Type B2: revision arthroplasty Versus Open reduction and internal fixation with locking Compression plate. Geriatr Orthop Surg Rehabil 10:2151459319876859. 10.1177/2151459319876859 31579528 10.1177/2151459319876859 PMC6759715

[22] Concina C, Crucil M, Gherlinzoni F (2021) Factors influencing results and complications in proximal periprosthetic femoral fractures: a retrospective study at 1- to 8-year follow-up. Acta Biomed 92:e2021022. 10.23750/abm.v92iS3.11734 34313661 10.23750/abm.v92iS3.11734 PMC8420837

[23] Lindahl H, Garellick G, Regnér H et al (2006) Three hundred and twenty-one periprosthetic femoral fractures. J Bone Joint Surg Am 88:1215–1222. 10.2106/JBJS.E.00457 16757753 10.2106/JBJS.E.00457

[24] Martinov S, D’ulisse S, Haumont E et al (2022) Comparative study of Vancouver type B2 periprosthetic fractures treated by internal fixation versus stem revision. Arch Orthop Trauma Surg 142:3589–3597. 10.1007/s00402-021-03953-6 33993361 10.1007/s00402-021-03953-6

[25] Slullitel PA, Garcia-Barreiro GG, Oñativia JI et al (2021) Selected Vancouver B2 periprosthetic femoral fractures around cemented polished femoral components can be safely treated with osteosynthesis. Bone Joint J 103–B:1222–1230. 10.1302/0301-620X.103B7.BJJ-2020-1809.R1 34192924 10.1302/0301-620X.103B7.BJJ-2020-1809.R1

[26] Flury A, Hasler J, Pagenstert G et al (2021) Open reduction and internal fixation might be a valuable alternative to stem revision in Vancouver B2 periprosthetic femoral fractures, irrespective of the stem’s design. Arch Orthop Trauma Surg 141:871–878. 10.1007/s00402-020-03568-3 32778919 10.1007/s00402-020-03568-3

[27] Powell-Bowns MFR, Oag E, Ng N et al (2021) Vancouver B and C periprosthetic fractures around the cemented Exeter Stem: sex is associate with fracture pattern. Bone Joint J 103–B:309–320. 10.1302/0301-620X.103B2.BJJ-2020-0695.R1 34390386 10.1007/s00402-021-04113-6 PMC9522678

[28] González-Martín D, Pais-Brito JL, González-Casamayor S et al (2021) Periprosthetic hip fractures with a Loose Stem: open reduction and internal fixation Versus Stem Revision. J Arthroplasty 36:3318–3325. 10.1016/j.arth.2021.05.003 34052099 10.1016/j.arth.2021.05.003

[29] Zheng H, Gu H, Shao H et al (2020) Treatment and outcomes of Vancouver type B periprosthetic femoral fractures: a retrospective study of 97 cases. Bone Joint J 102–B:293–300. 10.1302/0301-620X.102B3.BJJ-2019-0935.R1 32114805 10.1302/0301-620X.102B3.BJJ-2019-0935.R1

[30] Barghi A, Hanna P, Merchan N et al (2022) Outcomes after operative fixation of Vancouver B2 and B3 type periprosthetic fractures. J Orthop Trauma 36:228–233. 10.1097/BOT.0000000000002277 34581700 10.1097/BOT.0000000000002277

[31] Jain S, Farook MZ, Aslam-Pervez N et al (2023) A multicentre comparative analysis of fixation versus revision surgery for periprosthetic femoral fractures following total hip arthroplasty with a cemented polished taper-slip femoral component. Bone Joint J 105–B:124–134. 10.1302/0301-620X.105B2.BJJ-2022-0685.R1 36722066 10.1302/0301-620X.105B2.BJJ-2022-0685.R1

[32] Powell-Bowns MFR, Oag E, Martin D et al (2022) Vancouver B and C periprosthetic fractures around the cemented Exeter Stem: sex is associate with fracture pattern. Arch Orthop Trauma Surg 142:3221–3228. 10.1007/s00402-021-04113-6 34390386 10.1007/s00402-021-04113-6 PMC9522678

[33] Parker MJ, Palmer CR (1993) A new mobility score for predicting mortality after hip fracture. J Bone Joint Surg Br 75:797–798. 10.1302/0301-620X.75B5.8376443 8376443 10.1302/0301-620X.75B5.8376443

[34] Harris WH (1969) Traumatic arthritis of the hip after dislocation and acetabular fractures: treatment by mold arthroplasty. An end-result study using a new method of result evaluation. J Bone Joint Surg Am 51:737–755 5783851

[35] Lewis DP, Tarrant SM, Cornford L, Balogh ZJ (2022) Management of Vancouver B2 periprosthetic femoral fractures, Revision Total Hip Arthroplasty Versus Open reduction and internal fixation: a systematic review and Meta-analysis. J Orthop Trauma 36:7–16. 10.1097/BOT.0000000000002148 33942785 10.1097/BOT.0000000000002148

[36] Haider T, Hanna P, Mohamadi A et al (2021) Revision arthroplasty Versus Open reduction and internal fixation of Vancouver Type-B2 and B3 periprosthetic femoral fractures. JBJS Rev 9. 10.2106/JBJS.RVW.21.00008 10.2106/JBJS.RVW.21.00008 34415859

[37] González-Martín D, Hernández-Castillejo LE, Herrera-Pérez M et al (2023) Osteosynthesis versus revision arthroplasty in Vancouver B2 periprosthetic hip fractures: a systematic review and meta-analysis. Eur J Trauma Emerg Surg 49:87–106. 10.1007/s00068-022-02032-8 35790555 10.1007/s00068-022-02032-8

[38] Voeten SC, Nijmeijer WS, Vermeer M et al (2020) Validation of the fracture mobility score against the Parker mobility score in hip fracture patients. Injury 51:395–399. 10.1016/j.injury.2019.10.035 31668574 10.1016/j.injury.2019.10.035

[39] Nilsdotter A, Bremander A (2011) Measures of hip function and symptoms: Harris Hip score (HHS), hip disability and osteoarthritis outcome score (HOOS), Oxford Hip score (OHS), Lequesne Index of Severity for Osteoarthritis of the hip (LISOH), and American Academy of Orthopedic Surgeons (AAOS) hip and knee questionnaire. Arthritis Care Res (Hoboken) 63(Suppl 11):S200–207. 10.1002/acr.20549 22588745 10.1002/acr.20549

[40] Zuurmond RG, van Wijhe W, van Raay JJAM, Bulstra SK (2010) High incidence of complications and poor clinical outcome in the operative treatment of periprosthetic femoral fractures: an analysis of 71 cases. Injury 41:629–633. 10.1016/j.injury.2010.01.102 20236641 10.1016/j.injury.2010.01.102

[41] Moreta J, Uriarte I, Ormaza A et al (2019) Outcomes of Vancouver B2 and B3 periprosthetic femoral fractures after total hip arthroplasty in elderly patients. HIP Int 29:184–190. 10.1177/1120700018772163 29716387 10.1177/1120700018772163

[42] Papagrigorakis E, Galanis A, Vlachos C et al (2022) Periprosthetic fracture of total hip replacement in patients with osteopetrosis: a case presentation and review of literature. J Frailty Sarcopenia Falls 7:251–256. 10.22540/JFSF-07-251 36531518 10.22540/JFSF-07-251 PMC9729755

[43] Morgan S, Bourget-Murray J, Garceau S, Grammatopoulos G (2023) Revision total hip arthroplasty for periprosthetic fracture: epidemiology, outcomes, and factors associated with success. Annals Joint 8. 10.21037/aoj-23-16 10.21037/aoj-23-16 PMC10929400 38529253

[44] Kennedy JW, Hrycaiczuk A, Ng NYB et al (2022) Cement-in-cement versus uncemented modular stem revision for Vancouver B2 periprosthetic fractures. J Orthop 31:124–128. 10.1016/j.jor.2022.03.008 35541568 10.1016/j.jor.2022.03.008 PMC9079639

